# Language exposure, input diversity and receptive vocabulary affect stop and vowel perception in Spanish-dominant Spanish-English bilingual children

**DOI:** 10.1093/chidev/aacag084

**Published:** 2026-11-12

**Authors:** Simona Montanari, Jeremy Steffman, Robert Mayr

**Affiliations:** 1 Department of Child and Family Studies, California State University, Los Angeles, United States; 2 Centre for Speech, Hearing and Communication Research, Cardiff Metropolitan University, Cardiff, Wales, U.K; 3 Department of Linguistics and English Language, University of Edinburgh, Edinburgh, Scotland, U.K

**Keywords:** speech perception, bilingual children, Spanish and English

## Abstract

Language-specific speech perception intensifies at preschool age; yet no study has examined this phenomenon in bilinguals. This study explores stop and vowel perception in English and Spanish in 74 Mexican-origin children (mean age: 50 months; 37 Spanish-English bilinguals, 24 females; 37 English monolinguals, 23 females) and examines whether language exposure, input diversity, and receptive vocabulary moderate bilinguals’ perception in each language. Bilinguals’ English stop—but not vowel—perception, as assessed through a forced-choice minimal-pair identification task in 2022 and 2023, was partially affected by Spanish, suggesting subtle bilingual–monolingual differences. The effect of input diversity on perception was mediated by vocabulary in English and exposure in Spanish, highlighting the complex interplay of input- and proficiency-related variables on bilingual perception.

## Lay summary

This study examines how Spanish-English bilingual children hear the vowels and consonants of English and Spanish, and whether children’s ability to hear speech sounds in each language is related to language exposure, vocabulary size, and the number of people who speak to them in that language. Our findings show that the bilingual children differed only minimally from monolinguals in how they heard English sounds, and this was due to the influence of Spanish. Children’s ability to hear speech sounds was related to language exposure and vocabulary, and children with more exposure and larger vocabularies benefited from hearing that language from more speakers. These results have educational implications for the growing Spanish-English bilingual child population.

The process of speech perception, the ability to perceive linguistic structure in the acoustic speech signal, begins to emerge during the first year of life when infants transition from a phase of universal sound discrimination to a phase of increased discrimination of the sounds of their native language (Kuhl, et al., 2006; Werker & Tees, 1984). While this process begins in infancy, speech perception abilities continue to develop throughout childhood, with children’s speech sound categories becoming more categorical and well-defined with improved speech production abilities, larger vocabularies, and schooling (Hazan & Barrett, 2000; Nittrouer, 2002; Walley & Flege, 1999). Indeed, speech perception skills particularly mature between 3 and 5 years of age, when children learn to make correspondences between speech sounds and letters in order to develop literacy skills, and speech perception acuity is related to both prereading skills, such as the ability to identify and manipulate individual sounds in spoken words (i.e., phonemic awareness), and later reading outcomes (Snowling et al., 2019).

The majority of children around the world grow up in multilingual communities, so they face the additional challenge of extracting the speech sounds of each language from more varied linguistic input. Studies of both bilingual infants and children, whose speech perception development is dependent on sufficient exposure to the different sounds of each language as well as on the distinct frequencies and distributions of these sounds in the speech signal, have documented differences from monolingual patterns despite regular bilingual experience (Bosch & Sebastián-Gallés, 2003; McCarthy et al., 2014; Montanari et al., 2023; Ramon-Casas et al., 2023). However, the extent to which the ability to perceive different vowels and consonants differs for monolingual and bilingual children between 3 and 5 years of age and the factors that contribute to bilingual perceptual performance remain underexplored. Here, we investigate the similarities and differences in the perception of English stops and vowels for monolingual and Spanish-dominant Spanish-English bilingual children. We further examine the extent to which a host of child-internal and external variables—including language exposure, input diversity, and receptive vocabulary—are related to bilingual children’s perceptual abilities in both the societal and heritage language.

## The development of phonemic categories in two languages

During the first year of life, infants develop a heightened sensitivity to the speech sounds of their native language (Werker & Tees, 1984). This developmental pattern has been explained by attunement theories of development, which posit that infants, born to discriminate phonetic differences that are and are not part of their native language’s phonetic inventory, can differentiate both native and non-native similar-sounding speech sounds in the first half of the first year. Native language experience functions to tune, maintain, and facilitate the perception of native language phonemes, while resulting in a decline in non-native speech sound discrimination (Kuhl, et al., 2006; Werker & Tees, 1984). This process of perceptual attunement, which is dependent on perceptual exposure to native relative to non-native phonemic contrasts, occurs around 4–6 months for vowels and 10–11 months for consonants (Polka & Bohn, 2011; Polka & Werker, 1994; Stager & Werker, 1997; Werker & Tees, 1984).

Bilingual infants learning prosodically distant languages that can be distinguished at birth (like Spanish and English) have been shown to discriminate vowel and consonant contrasts in both languages similarly to monolingual peers, even those similar sounding phonemes that are not produced in exactly the same way (see Sundara, 2024, for a review). However, bilingual infants learning prosodically-similar languages that cannot be distinguished at birth (like Spanish and Catalan) demonstrate a different pattern of development, with a temporary delay in their attunement to native categories that are acoustically similar but map differently to their two languages (Bosch & Sebastián-Gallés, 2003). This U-shaped developmental trajectory has also been evidenced in neuroimaging studies (Ramirez et al., 2017), suggesting that learning speech sounds in prosodically-similar languages is a difficult task and bilingual infants may require additional accumulated exposure to their two languages in order to track and learn the competing distributional properties of speech sound categories.

Research on speech perception in older bilingual children is scarce, but it confirms that 4- and 5-year-old bilinguals may display perceptual patterns that differ from monolingual patterns despite years of experience and regular use of each language. For example, McCarthy et al. (2014), who assessed English stop voicing perception in Sylheti L1-English L2 bilingual children growing up in London, found that perception patterns were different from those of English-speaking monolinguals and were affected by children’s Sylheti phonemic categories at age 4;4. Ramon-Casas et al. (2023) similarly documented better performance for Catalan-dominant 4.5-year-old bilingual children with respect to Spanish-dominant ones in identifying correct and mispronounced words containing the /e-ɛ/contrast, which is phonemic in Catalan but not in Spanish. Montanari et al. (2023) also found that Spanish-English bilingual children’s category boundaries for English /b–p/ and /d–t/ were positioned at lower voice onset time (VOT) values as compared to those of English monolingual peers, consistent with the lower VOT values that characterize the Spanish /b–p/ and /d–t/ categorical boundaries. This was the case even if the bilingual children heard more English than Spanish. Taken together, these studies suggest that, in bilingual development, phonemic representations in one language may influence those in the other language leading to differences from monolingual patterns.

Nonetheless, phonemic categories can be acquired and refined with language experience. Indeed, after an additional year of English experience in preschool, the bilinguals in McCarthy et al. (2014) displayed English perceptual patterns that no longer differed from those of English-speaking monolinguals. The children were now 5;4 of age, a time in which children reach a peak in language-specific speech perception (Horlyck et al., 2012). Montanari et al. (2023) also found more mature English /b-p/ perception for 5-year-olds than for 4-year-olds. In contrast, Montanari et al. (2024) did not find bilingual–monolingual differences in the perception of the English /i-ɪ/ vowel contrast for the same participants. The authors interpreted this finding as evidence of the earlier emergence of native vowel perception (in line with the earlier vowel attunement that occurs in the first year of life, Polka & Werker, 1994) or as an indication of the more limited susceptibility to cross-linguistic interaction for this vowel contrast. Thus, it is unclear whether vowel and consonant perception differ for monolingual and bilingual children between 3 and 5 years of age. Also, bilingual children’s perception has been primarily studied only in the societal language; therefore, the full range of bilingual children’s perception abilities—and the extent to which children’s perceptual skills in each language interact in development—remain underexplored.

## Factors that contribute to the development of language-specific speech perception

While the canonical view on speech perception is that children attune to the sounds of their native language(s) within their first year of life, there is mounting evidence that children continue to develop speech sound categories throughout childhood, when they experience significant changes in general cognition as well as improvements in speech production and growth in vocabulary (Hazan & Barrett, 2000; Nittrouer, 2002). The preschool and kindergarten years are particularly important as the monolingual literature has shown that children’s perceptual attunement to native over non-native sounds is heightened between 4 and 6 years of age (over and above the attunement established in infancy), and the degree of this heightened perceptual attunement predicts reading and reading-related phonological skills (Burnham et al., 1991; Horlyck et al., 2012). Specifically, Burnham et al. (1991) found an improvement in the perception of a native contrast from infancy to adulthood, with an accelerated increase between 2 and 6 years, when children experienced a peak in language-specific perception that overlapped with the emergence of reading abilities. At the same time, however, the children experienced a marked dip in nonnative speech perception between these ages, suggesting that perceptual narrowing to native speech sounds occurs at the expense of more global sensitivity to all speech variations. These results make it interesting to study the intensification of language-specific speech perception in bilingual children who are exposed to heterogeneous as well as reduced input in each of their languages.

The limited research with this population has shown that certain input characteristics, such as input quantity and input diversity, may be related to how well children can perceive the sounds of their languages. As can be expected, input quantity—or the amount of exposure a child receives to a language—is positively linked to perceptual performance in that language. In McCarthy et al. (2014), it took children almost two years of regular and consistent exposure to English in preschool in order for them to attain English perceptual patterns that did not differ from those of English-speaking monolinguals. Ramon-Casas et al. (2023) also found that the children with higher exposure to Catalan reliably outperformed the children who heard more Spanish in their perception of the Catalan /e-ɛ/ contrast. Yu et al. (2019) further demonstrated that language exposure moderated vowel discrimination between 3 and 47 months of age, with only bilingual children with higher English exposure processing the English contrast similarly to monolinguals. These studies, however, assessed perceptual performance only in the societal language. Montanari et al. (2023) found that language exposure solely predicted perceptual performance in Spanish, the heritage language, whereas it did not affect English perception. Thus, while the findings suggest, overall, that higher language exposure leads to better perceptual abilities, it is unclear whether the amount of input that is needed to develop native-like perception varies for the heritage and societal language and for the dominant vs. non-dominant language.

An additional input characteristic that may be linked to perceptual performance is input diversity, that is, the number of input providers that children have in each language. Bilingual and monolingual research has found that multiple-talker input is more supportive of lexical and grammatical development than input from fewer speakers (Huttenlocher et al., 2010; Place & Hoff, 2011, 2016). With respect to speech perception, Montanari et al. (2023) failed to document a relation between input diversity and consonant perception in either the societal or heritage language. However, Montanari et al. (2024) found that input diversity interacted with input quantity to mediate the perception of the English /i–ɪ/ vowel contrast in the same children, with diverse input promoting perceptual performance in children receiving higher English exposure while limiting perception in children with more limited English input. The authors speculated that input diversity may promote vowel perception at relatively high levels of language exposure—and more advanced stages of language learning, when children can possibly use input from multiple speakers to sample more of the vocalic space and refine their perceptual categories. However, at low levels of exposure and earlier stages of learning, diverse input may make it harder for children to distinguish between, and form, new perceptual categories, thus limiting perceptual performance. Clearly, more studies are needed to elucidate the role of input diversity on bilingual perception.

While the limited research on perception in bilingual children has focused on the impact of input-related variables (i.e., what children *hear*), no study to our knowledge has examined the role of receptive vocabulary (i.e., what children *know*) on perceptual performance, which can be thought of as a proxy of overall language skills. More advanced language skills coupled with a larger vocabulary (including knowledge of more phonologically similar words) have indeed been shown to increase learners’ sensitivity to fine-grained detail, forcing learners to develop more detailed phonological representations and enabling more discrete categorization (Metsala & Walley, 1998; Walley & Flege, 1999). Vance et al. (2009), for instance, found that monolingual 4- and 5-year-old children with larger vocabularies and better comprehension skills were also better at detecting mispronunciations of words in which one phoneme had been substituted. These results suggest that increases in vocabulary size lead children to make generalizations about the phonological structure of their language, a process that results, in turn, in more robust phonological representations. Since bilingual children may have different vocabulary sizes in each of their languages, they provide the ideal ground to examine the role of biological or maturational factors vs. proficiency in children’s intensification of language-specific perception.

## The present study

This study expands the literature on bilingual speech perception by examining the perceptual performance of Spanish-dominant Spanish-English bilingual children with both consonants and vowels in both English and Spanish during the intensification of language-specific speech perception that occurs between 3 and 5 years of age. Our first goal is to compare the bilinguals’ perception of the English /b-p/, /d-t/, and /i–ɪ/ contrasts to that of English-speaking monolingual peers to assess whether exposure to Spanish has consequences on English sound discrimination. We have limited the bilingual–monolingual comparisons to English because the participants are growing up in the United States and, in order to be successful in school and in life, they are expected to develop English perceptual skills that are on par with those of monolingual English-speaking peers, whereas comparing heritage language speakers to monolinguals in geographically-distant regions is methodologically flawed due to evident input and language learning environment differences (Bayram et al., 2021). Nonetheless, in order to examine the full range of bilingual children’s perception abilities, our second analysis examines the extent to which input quantity, input diversity, and receptive vocabulary moderate perception patterns in both English and Spanish. Specifically, we examine the role of caregiver-reported English exposure, the number of English input sources, and English receptive vocabulary in moderating bilingual children’s English perception patterns. Likewise, we investigate the links between caregiver-reported Spanish exposure, the variety of Spanish input sources, and receptive Spanish vocabulary and Spanish perception.

**Figure 1:**
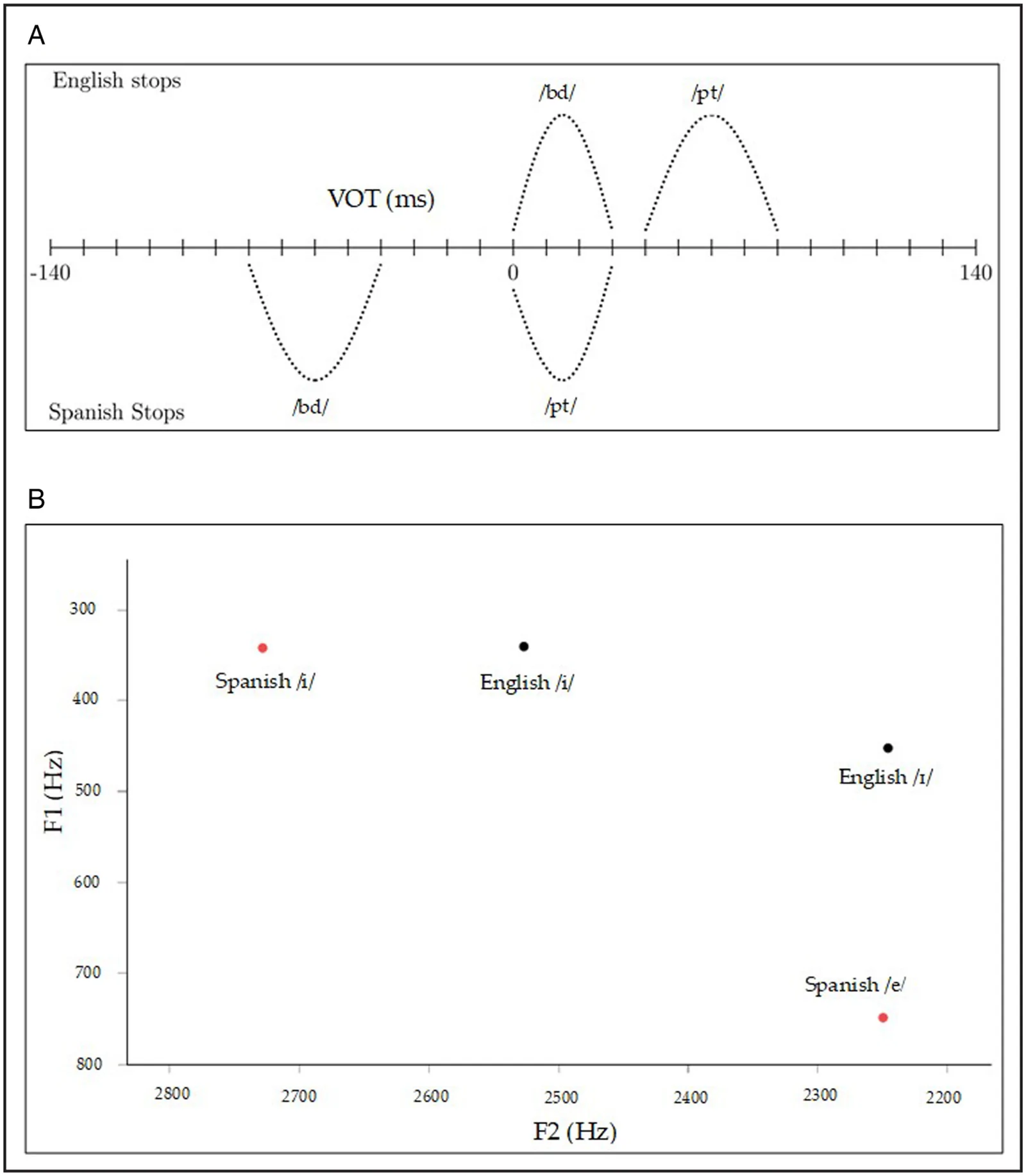
Schematization of the stop contrasts (panel A) and vowel contrasts (panel B) in English and Spanish examined in the present study.

We focus on the consonant contrasts /b–p/ and /d–t/ since both English and Spanish contrast voiced and voiceless categories, but their phonetic realizations differ between the two languages as assessed through voice onset time (VOT), i.e., the timing relation between the release of a stop and the onset of voicing on the following vowel: English uses long-lag VOT (i.e., aspiration) for voiceless stops and short-lag VOT for voiced ones, whereas Spanish uses short-lag VOT for voiceless stops and lead VOT (i.e., prevoicing) for voiced stops. Thus, English voiced stops acoustically overlap with Spanish voiceless stops, posing a challenge for their acquisition both in perception (Montanari et al., 2023) and production (Mayr et al., 2025). This language-specific difference in the implementation of the voicing contrast is schematized in Figure 1 (Panel A). Besides being susceptible to cross-linguistic interaction, the development of the voicing contrast in English and Spanish is also affected by developmental factors, with the English voicing contrast being developed before the one in Spanish, at least in production, due to the aerodynamic challenges that prevoicing poses (Macken & Barton, 1980a, 1980b). Thus, Spanish-English bilingual children face the double task of having to manage both developmental and cross-linguistic interaction challenges when acquiring the voicing contrast in both languages. Note that we focus on stop perception at two places of articulation, bilabials and coronals—thus excluding velars, in order to strike an optimal balance between examining place of articulation effects and making the task feasible for young children in terms of duration and trial count. While we originally pilot tested a task that assessed perception across all places of articulation, the children failed to participate in a meaningful way due to the length of the test. Moreover, English and Spanish stimuli that were matched for following vowel contexts and syllable counts were more readily available for bilabials and coronals than for velars (see “Materials” below). Hence, similarly to other perception studies in young populations (McCarthy et al., 2014; Ramon-Casas et al., 2023), we limited our study to two rather than all three stop categories.

We also assess the perception of the /i–ɪ/ vowel contrast in English and of the /i-e/ vowel contrast in Spanish, with all four vowels constituting monophthongs with steady-state vocalic trajectories. We focus on /i–ɪ/ in English because extensive work demonstrates that L1 Spanish listeners—even early Spanish–English bilinguals—may perceive both /i/ and /ɪ/ as exemplars of the same Spanish category /i/ and therefore fail to differentiate the two English vowels (Baigorri et al., 2019). At the same time, recent work has shown that Spanish-English bilingual children who heard more English than Spanish perceived the /i–ɪ/ contrast similarly to English monolingual peers (Montanari et al., 2024). Since these participants were more exposed to English than Spanish, it remains to be seen whether Spanish-dominant bilingual children perceive this contrast similarly or differently from monolingual peers. The most critical acoustic dimensions on which English /i/ and /ɪ/ differ are the first and second formant frequencies (F1 and F2), with /i/ being characterized by lower F1 values and higher F2 values than /ɪ/, indicating a closer and more fronted vowel quality. In addition, English /i/ is intrinsically longer than /ɪ/, with duration acting as a secondary cue to vowel identity (Hillenbrand et al., 1995). Furthermore, we focus on the Spanish vowel contrast /i-e/ in order to assess vowel perception in the heritage language. We acknowledge that the English and Spanish vowel contrasts are different, limiting cross-language comparisons. Nonetheless, using a Spanish vowel contrast that covers a similar area of the vocalic space as English /i–ɪ/ was deemed appropriate to examine the extent to which child-internal and external variables moderated perceptual abilities *separately* in each language. Spanish /i/ and /e/ are primarily distinguished in terms of F1 and F2, with lower F1 and higher F2 values for /i/ than /e/, and little difference in duration. Figure 1 (panel B) depicts the F1 and F2 values of the natural vowel productions of English /i-ɪ/ and Spanish /i-e/ that acted as the endpoints in the vowel continua used in the present study. As the figure shows, the Spanish contrast is spectrally more distinct than the English one.

We ground our study in Flege’s Speech Learning Model-revised (SLM-r, Flege & Bohn, 2021), which proposes that when L2 phones are similar to those of the L1, L1 categories act as attractors and L1 and L2 sounds may form merged representations that have compromise values that differ from those of monolinguals. We also ground our study in Best’s L2 Perceptual Assimilation Model (PAM-L2; Best & Tyler, 2007), according to which L2 contrasts that are assimilated to a single native category are particularly hard to discriminate perceptually. While these models were proposed for L2 adult learners, they are the most influential speech perception models and can also account for the perceptual performance of children who are learning two languages early in life. We thus hypothesize, based on the SLM-r and PAM-L2 predictions as well as on the results of the studies reviewed above, that the bilingual children will have more difficulty with the perception of English voiced stops than monolinguals since voiced stops in English acoustically overlap with voiceless stops in Spanish. However, given the mixed findings regarding /i–ɪ/ perception in child and adult Spanish-English bilinguals, we are unable to put forward a conclusive hypothesis as to whether English vowel perception will differ for the bilingual and monolingual children. With respect to the moderator variables, we speculate, based on the results of the studies reviewed above, that language exposure, receptive vocabulary and input diversity will be linked to children’s perceptual performance. Yet, due to the literature’s mixed findings, we are unable to predict the role of each variable in Spanish and English.

## Method

### Participants

The study was approved by the Institutional Review Board of the university in which the study was conducted (project #1796206–1), and before data collection, the parents of the child participants provided written informed consent. A total of 74 children participated in the study. Of these, 37 (24 girls, 13 boys) were Spanish-English bilinguals, while the remaining 37 (23 girls, 14 boys) were English monolinguals. The children were recruited from a bilingual community in Southern California with a 94.5% concentration of Latinx residents the vast majority of whom (83.5%) are Spanish speakers (https://data.census.gov/). Recruitment and testing were conducted by trained Child Development students as part of a language development course at a public, 4-year urban university in 2022 and 2023. Only children with no reported history of speech, language, hearing, cognitive, or neurological issues were included. The bilingual children had a mean age of 51.16 months (SD = 9.55), which did not significantly differ from the monolinguals’ mean age of 48.95 months (SD = 7.41; *t*(73) = 1.099, *p* = .138). Additionally, there were no significant differences in the gender distribution between the bilingual and monolingual groups (χ^2^ = .058, *p* = .809). Both groups were matched in socioeconomic status (SES), as all children came from low-to-middle-income families in the same community. The bilingual children were of Mexican heritage; they had been raised in homes in which they heard both Spanish and English through family members and were active users of both languages. The monolingual children were also of Mexican descent and part of the same Latinx community, exposed to Spanish in the broader environment. However, as they were primarily exposed to English at home and did not actively use or understand Spanish, as assessed in the lab through a conversation with a research assistant, they were classified as functional monolinguals.

Table 1 presents a summary of the children’s language exposure and proficiency, including the amount of English and Spanish input they were exposed to, the number of input sources for each language, and English and Spanish receptive vocabularies. Language exposure was reported by parents using a scale from 1 to 5, with 1 = *child hears mostly Spanish*, 2 = *child hears more Spanish than English*, 3 = *child hears as much Spanish as English*, 4 = *child hears more English than Spanish*, and 5 = *child hears mostly English*, with higher scores thus representing higher exposure to English. While measures of absolute language exposure (i.e., 12 hr of exposure to Spanish and 1 hr to English) may better capture the number of hours a child hears a language, time-efficient and parent-friendly measures of relative language exposure like ours have been shown to reliably estimate a child’s overall bilingual experience (Calandruccio et al., 2021). Based on this scale, the bilingual children’s average exposure score was 2.86 (SD = 0.87, range: 1–4), while that of the monolingual children was 4.7 (SD = 0.46, range: 4–5), a difference that was statistically significant (*t*(73) = 11.171, *p* < .001), confirming that the latter were significantly more exposed to English than the former.

Input diversity in each language was assessed by asking parents to report which individuals spoke Spanish and English to their child, with the option to list multiple sources, including “mother, father, siblings, grandparents, babysitter, teacher, media, and other” (allowing for 1 to 8 sources). While we could not assess the amount of input provided by each source, we speculated that more input sources provided more opportunities to hear the different sounds, words, and sentences of each language, increasing the diversity and thus the quality of the input (Huttenlocher et al., 2010; Place & Hoff, 2011, 2016). Based on this information, bilingual children were exposed to an average of 4.14 input sources in Spanish (SD = 1.41, range: 1–6) and 3.78 input sources in English (SD = 0.93, range: 1–5), with the difference between the two not being statistically significant (*t*(72) = 1.199, *p* > .05). In contrast, the English monolingual children heard English through an average of 5.08 input sources (SD = 0.75, range: 4–7). Bilinguals and monolinguals differed on the number of English sources (*t*(72) = 6.50, *p* > .001), indicating that the bilingual children were exposed to English from fewer speakers compared to the monolinguals.

The participants’ receptive English and Spanish vocabularies were assessed by the *Peabody Picture Vocabulary Test*, 5th ed. (PPVT-5, Dunn, 2018) in English and the corresponding Spanish *Test de Vocabulario en Imágenes Peabody* (TVIP, Dunn et al., 1986) in Spanish. The monolinguals were only assessed in English. On these standardized tasks, children select an image from an array of four pictures that best matches the word spoken by the experimenter. Children’s raw score is the number of correct choices before reaching ceiling. Standard scores are based on monolingual norms, with a mean of 100 and standard deviation of 15. Note that standard scores are given to show the extent to which our typically developing bilingual participants had vocabulary sizes commensurate to those same-age monolingual peers and to compare English and Spanish vocabulary sizes. However, raw scores were used as the moderator variables in the analyses, as in similar studies (cf. Paradis & Jia, 2017; Sorenson Duncan & Paradis, 2020). The bilingual children’s mean raw PPVT score was 71.05 (SD = 29.33, range: 20–126), and their mean standard score was 91.81 (SD = 12.64, range: 68–118). In contrast, the monolingual children’s mean raw PPVT score was 86.32 (SD = 24.6, range: 39–135), and their mean standard score was 104.62 (SD = 15.4, range: 80–160). Mean PPVT standard scores were significantly different for the bilinguals and monolinguals (*t*(72) = 3.557, *p* > .001), suggesting that the monolinguals had significantly larger English vocabulary sizes than the bilingual children. However, the bilingual children also knew a considerable number of Spanish words: their mean raw TVIP score was 27.76 (SD = 13.86, range: 7–56) (the TVIP includes approximately half of the number of items that are in the PPVT), and their mean TVIP standard score was 103.24 (SD = 16.77, range: 63–140). Mean PPVT and TVIP standard scores were significantly different for the bilinguals (*t*(35) = 3.117, *p* > .01), suggesting that the children knew significantly more words in Spanish than in English and thus were dominant in Spanish.

### Materials

To assess the children’ perception of English and Spanish bilabial and coronal stops, as well as the English /i–ɪ/ and Spanish /i-e/ vowel contrasts, we created a child-friendly forced-choice minimal-pair picture identification (2AFC) task in each language that we ran through Qualtrics (Qualtrics, 2022). In each task, children listened to an auditory stimulus that varied systematically along the VOT and vowel continuum and were asked to match it to one of two pictures representing a minimal pair. For the English task, we used the minimal pairs *penny/Benny, toe/doe* and *sheep/ship*. For the Spanish task, we used the minimal pairs *peso* (i.e., Mexican currency)/*beso* (“kiss”), *tos* (“cough”)/*dos* (“two”) and *piso* (“floor”)/*peso*. We selected these words for the bilabial and coronal stops because they were matched between Spanish and English in terms of number of syllables and vowel context. The items for the vowel contrasts were not matched between languages, as the /i–ɪ/ and /i-e/ contrasts are distinct and were intended to assess only language-specific perception without cross-language comparison. The selected words were chosen for their imageability and ease of teaching to children, even if they were not already familiar with them. Although some of these words are more frequent than others, we ensured that children were familiarized with the words one week before the experiment (see “Procedure” for details).

We then created six phonetic continua ranging from *penny* to *Benny*, *toe* to *doe*, and *sheep* to *ship* in English, and *peso* to *beso*, *tos* to *dos*, and *piso* to *peso* in Spanish. The stimuli were recorded by two different female speakers, each a native speaker of American English and Mexican Spanish, respectively. The Mexican Spanish speaker had been in the U.S. for less than six months and had limited knowledge of English, minimizing the likelihood of cross-language interaction. The American English speaker was not a Spanish speaker. Both speakers were recorded in a quiet environment using a Yeti Blue USB Microphone for English and a Tascam DR-07X for Spanish. The recordings were then digitized at 44.1 kHz and 32 bit depth.

VOT was manipulated using a Praat script (Winn, 2020) and the “cutback and replacement” approach described in Winn (2020), which increases the duration of voice onset time and simultaneously decreases the duration of the following vowel, given the well-documented inverse relation between the duration of these two cues (e.g., Allen & Miller, 1999). As is required in the “cutback and replacement” method, the starting tokens for manipulation in each case were words with initial /b/ and /d/ (in each language), for which the vowel was cut and replaced with aspiration noise to create /p/ and /t/. Vowel duration thus varied in these stimuli too, although we assumed that VOT would be the primary cue to the contrast, as shown for Spanish-English bilinguals perceiving English stops (Flege & Efting, 1987; Schertz et al., 2020; Toscano & McMurray, 2012).

We set the VOT endpoint values for the continua based on approximate ranges for VOT between voiced and voiceless stops in each language. We opted to use the same VOT range (90 ms), but to locate it differentially along a VOT continuum for each language, corresponding to what we predicted would be good exemplars of voiced and voiceless stops based on previous speech production research which shows VOT norms for both languages (e.g., Dmitrieva et al., 2015). For Spanish, the range was −40 (pre-voiced) to 50 ms VOT. For English, the range was 0 to 90 ms VOT. For each language, we used the same range for each place of articulation. We opted to create language-specific ranges because both Spanish voiceless stops with long-lag VOT and English voiced stops with substantial pre-voicing may sound unnatural to listeners. Each continuum was created to have 10 equidistant VOT steps within each language’s range.

Manipulation of the vowel continuum was carried out using a Praat script, employing the Burg Method of LPC decomposition and resynthesis (Winn, 2019). The formant values for each end point of the continuum were based on the naturally produced formants for English /i/ and /ɪ/ and Spanish /i/ and /e/. The resynthesis process estimated source and filter models for one end point (the word with /i/). The filter model’s F1, F2, and F3 were then interpolated linearly (in Bark space; Traunmüller, 1990) to the values of the other continuum end point, resulting in 10 intermediate filter steps. Phase-locked higher frequencies from the starting base file (/i/) that were lost in the process of LPC resynthesis were restored to all steps. The result was two 10-step continua ranging from /i/ to /ɪ/ and from /i/ to /e/. In English, we next manipulated vowel duration to co-vary with F1, F2, and F3 based on the speakers’ natural productions, whereby /i/ was longer than /ɪ/ (in agreement with large-scale studies measuring vowel duration; Hillenbrand et al., 1995). The duration manipulation thus interpolated between end point duration values in 10 linearly equidistant steps in the way that duration and vowel formants would normally co-vary (e.g., the /i/ end point had the longest duration, the /ɪ/ end point had the shortest duration). In Spanish, vowel duration was also varied to match the differences in duration between /i/ and /e/ in the speaker’s recordings; these differences were very small as compared to those in the English stimuli. In order to reduce the total number of stimuli presented, we next subset the six continua by excluding the next-to-endpoint steps from presentation, i.e., steps 2 and 9 from the range of steps 1–10 (see e.g., Kingston et al., 2016, for a similar approach). This trimming of the steps used allowed us to keep clear endpoints (steps 1 and 10), and sample more densely from ambiguous regions of the continuum (steps 3–8). There were thus a total of eight steps on each of the six continua presented to the child listeners.

### Procedure

The experiments were conducted in individual sessions in a university research laboratory. The children were asked to participate in a game in which a parrot had to learn new words. In a given trial, children heard an auditory stimulus and were asked to match it to one of two pictures that were presented on the screen so that the parrot could learn the new word. The placement (left vs. right) of the correct picture was counterbalanced across trials. The task was administered by a research assistant who played the auditory stimulus when the child was attentive and clicked on the picture selected by the child. There were a total of 24 trials in each experiment (8 for /b/-/p/, 8 for /d/-/t/ and 8 for /i/-/ɪ/ in English and 8 for /b/-/p/, 8 for /d/-/t/ and 8 for /i/-/e/ in Spanish). The trials were randomly presented, and, in order to maintain the child’s attention, they were interspersed with puzzles that gradually revealed a treat for the parrot. Each experiment took between 4 and 5 minutes to complete. Children were familiarized with the stimuli one week before the experiment. Parents were instructed to teach the stimuli to their child using the same pictures that we used in the experiment and to later send a video showing their child independently identifying each word. Children’s knowledge of each item was checked again in the lab before the experiment and only children who demonstrated 100% accuracy in identifying each test item were allowed to participate. Based on this criterion, all children could participate in the testing.

Both the English and Spanish data were collected in a single session in order to accommodate the families and avoid participant attrition. To set the bilingual participants in a monolingual mode (Grosjean, 2001), to the extent that this is possible in a bilingual experiment, English and Spanish data collection was maximally spaced out within a single session on the same day, with a change of experimenter (from an English-speaking to a Spanish-speaking one and vice versa) and a time set for play, conversation and verbal instructions in the target language before beginning the task. Half of the bilingual children completed the experiment in Spanish first, whereas the other half completed it in English first. The English monolingual children completed it only in English.

### Data analysis

All data, models, and analysis code are included online on the OSF: https://osf.io/w57ar/. We present several analyses in the results section. First, we consider how the bilingual and monolingual group compare to one another in their perception of the English stimuli. Secondly, for bilinguals, we examine how various moderator variables impact their perception of both English and Spanish stimuli. In this section, we describe the general modeling approach and methods, and then the specific model structure for each of the analyses. Note that our analyses are of an exploratory nature considering the novel domain of this research, the size of the sample, and the lack of clear initial hypotheses.

We modeled the data using Bayesian mixed-effects linear regression models fit with a logistic link function using brms (Bürkner, 2017). The dependent variable in all models was listeners’ response option (one of two choices presented on each trial). Two models were run to assess differences between bilingual and monolingual groups, one for categorization of the two stop contrasts and another for the vowel contrast, in all cases with the English stimuli. The stop model predicted listeners’ responses with a voiceless response (/p/ or /t/) mapped to 1 and a voiced response (/b/ or /d/) mapped to 0. Fixed effects in the model were VOT step (scaled and centered), group (bilingual mapped to −0.5, monolingual mapped to 0.5), and place of articulation (bilabial mapped to −0.5, and coronal mapped to 0.5). All interactions between fixed effects were included. Random effects were a random intercept for participant and random slopes for all within-participant effects (contrast and step). The model for the vowel contrast was the same in all respects, except it did not include a place of articulation variable as a fixed effect nor as a random effect. The moderator analyses for bilinguals only (hence with no group variable) were run in four models, one for each contrast type (stop vs. vowel) and each language. There were three further moderator variables in addition to continuum step (and place of articulation for the stop model): language exposure score (scaled and centered), number of input providers for the relevant language (scaled and centered), and vocabulary, i.e., PPVT raw scores for the English stimuli and TVIP raw scores for Spanish (scaled and centered). Interactions among each of these were also included in the fixed effects structure. The random effects were intercepts for participant and slopes for all within-participant fixed effects, that is, place of articulation for the stop models and scaled continuum step for all models. To confirm adequate sampling and model convergence, Bulk ESS, Tail ESS and R^ values were inspected. Additional details pertaining to model priors and sampling are included online in the modeling supplement on the OSF.

When reporting results, we provide the median posterior value for a particular estimate and the 95% credible intervals (CrI), which indicate the range of values in which 95% of the posterior distribution for that estimate falls. We also report the probability of direction metric, as computed with the package bayesterR (Makowski et al., 2019). When CrI excludes the value of zero, this means that the effect is estimated with a consistent directionality, i.e., mostly positive or negative (which will vary based on how variables are coded), and that, accordingly, the effect is likely to exist, with that particular directionality. Conversely, CrI that include the value of zero indicate variation in the estimated directionality of the effect. The PD metric is another way of characterizing the disposition of a posterior distribution and indicates the *percentage* of the distribution that shows a given directionality. In complementing CrI, pd is more intuitively mapped to probabilities of effect existence; for example, a pd value of 95 would indicate, according to the model, a 95% chance that the effect exists with that directionality. In presenting the results, we consider estimates with pd values of 95 or higher, though we note that this is an arbitrary threshold. Nevertheless, it presents a reasonable level of confidence in an effect and allows us to focus on those effects that are evidenced most robustly by the model. When twoway, or higher-order, interactions are considered, we visualize the estimates and use the estimate_slopes function from the model-based package (Makowski et al., 2020) to examine interactions involving a continuous variable.

We excluded data from participants who did not show sensitivity to the continuum, totaling 2.1% of the data. We reasoned that participants with zero slopes or slopes showing the opposite directionality of the standard expectation (i.e., more voiced responses with longer VOT) are either inattentive to the task or have categories that do not map to the canonical voiced/voiceless distinction. Methods like this are common in the speech perception literature as objective criteria for excluding participants (e.g., Buschong & Jaeger, 2019; Zhang & Steffman, 2025). This is described in more detail in the modeling supplement on the OSF. This exclusion was carried out before any analysis, and was not pre-registered.

## Results

### Comparison of English stop and vowel perception between bilinguals and monolinguals

**Figure 2:**
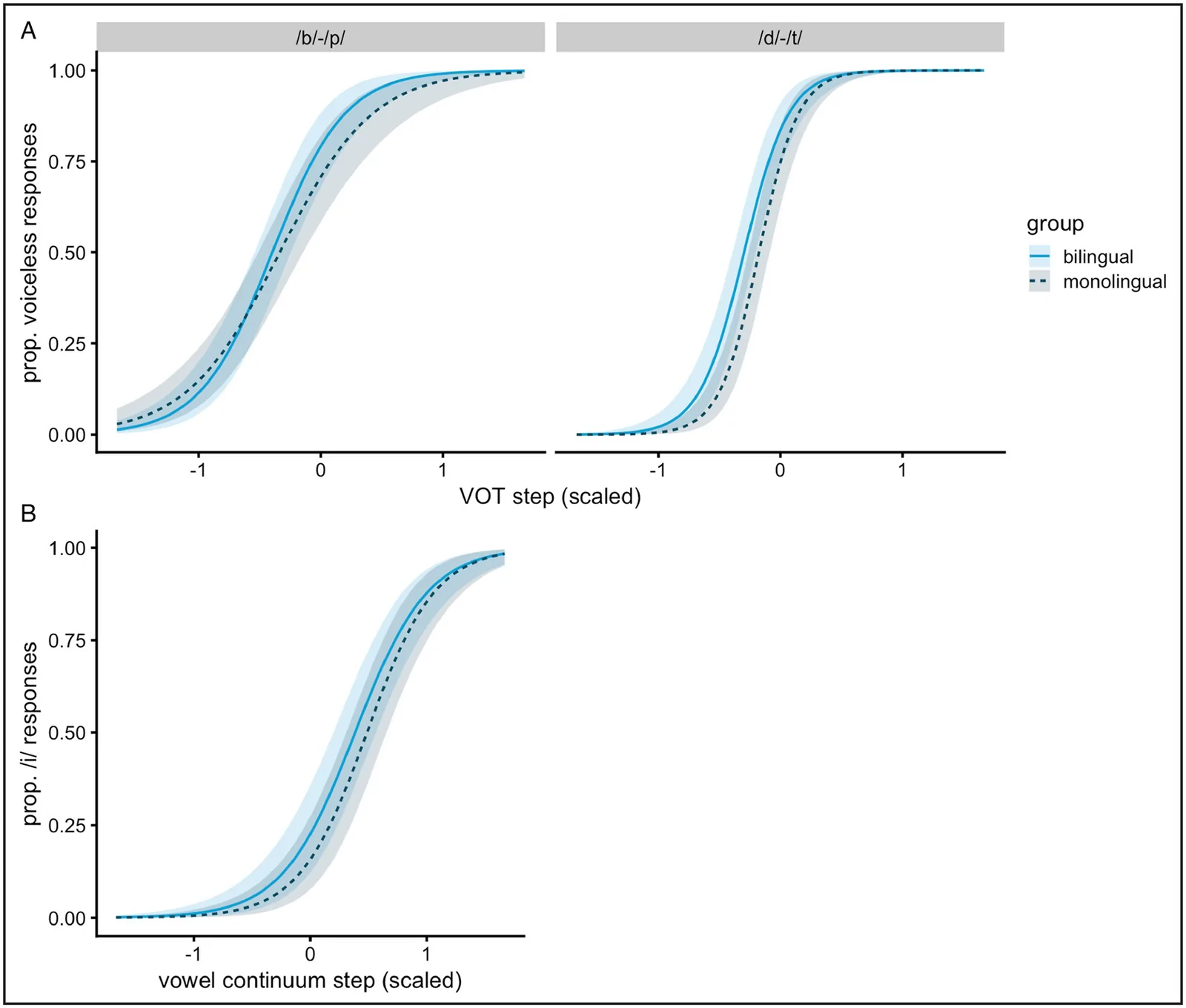
Model predictions for the stop model (panel A) and the vowel model (panel B). In the stop model, results are paneled by contrast. Line type and color indicate group. Ribbons are 95% credible intervals from the model.

Our principal interest in the first analysis is whether we have evidence for a group difference in the perception of both the English stop and vowel contrasts. Beginning with the model assessing perception of the stop contrasts, there was a credible effect of continuum step, as expected, showing that increasing the steps numerically along the continuum results in increased log-odds of a voiceless response (β = 4.46, CrI = [3.74,5.28], pd = 100). We found weak evidence for a main effect indicating a group-level difference (β = −0.50, CrI = [−1.15,0.12], pd = 94). This can be interpreted as a 94% chance that a group-level difference exists in this direction. The data are visualized in Figure 2A, where bilinguals’ categorization slopes are positioned more to the left as compared to the monolinguals’, indicating a lower category boundary and more voiceless responses. This pattern is in line with the fact that, in Spanish, positive VOT values (the whole English continuum) are mapped to the voiceless category, whereas English monolinguals map positive short lag VOT to a voiced category, thus giving overall fewer voiceless responses. However, given a PD value of 94, this effect should be interpreted cautiously.

There was also a credible interaction between contrast and continuum step (β = 3.04, CrI = [1.77,4.48], pd = 100). As can be seen in Figure 2A, this reflects the fact that categorization slopes are steeper for the coronal contrast (β = 6.16, CrI = [4.96,7.63]) than the bilabial contrast (β = 3.13, CrI = [2.50,3.94]), indicating steeper categorization for /d-t/ than /b-p/. There was additional evidence for a three-way interaction between continuum step, group, and contrast (β = 1.44, CrI = [−0.22,3.10], pd = 96). This interaction provides some additional evidence of bilingual–monolingual differences in /b-p/ perception, with the bilinguals’ providing more voiceless responses for the more ambiguous items in the middle of the continuum with respect to the monolinguals, while perceiving the VOT endpoint values similarly to the monolinguals. However, since the CrI narrowly includes zero, we should consider this interaction exploratory, as we can only be 96% sure that the interaction exists with this directionality.

Turning to the vowel model, for which estimates are shown in Figure 2B, there was the expected effect of continuum step (β = 3.35, CrI = [2.71,4.13], pd = 100); however, there was not strong evidence for a group level difference (β = −0.45, CrI = [−1.37,0.47], pd = 83), neither was there evidence for an interaction between step and group (pd = 68), leading us to conclude that the two groups perceive the vowel contrast broadly in a similar way.

### Moderators of bilingual children’s English stop and vowel perception

In the moderator analysis for English stops, there was both a main effect of continuum step, and a credible interaction with contrast. These findings replicate the results of the group analysis, showing steeper categorization for the coronal contrast, which can also be seen in the bilinguals’ categorization functions in Figure 2A.

**Figure 3:**
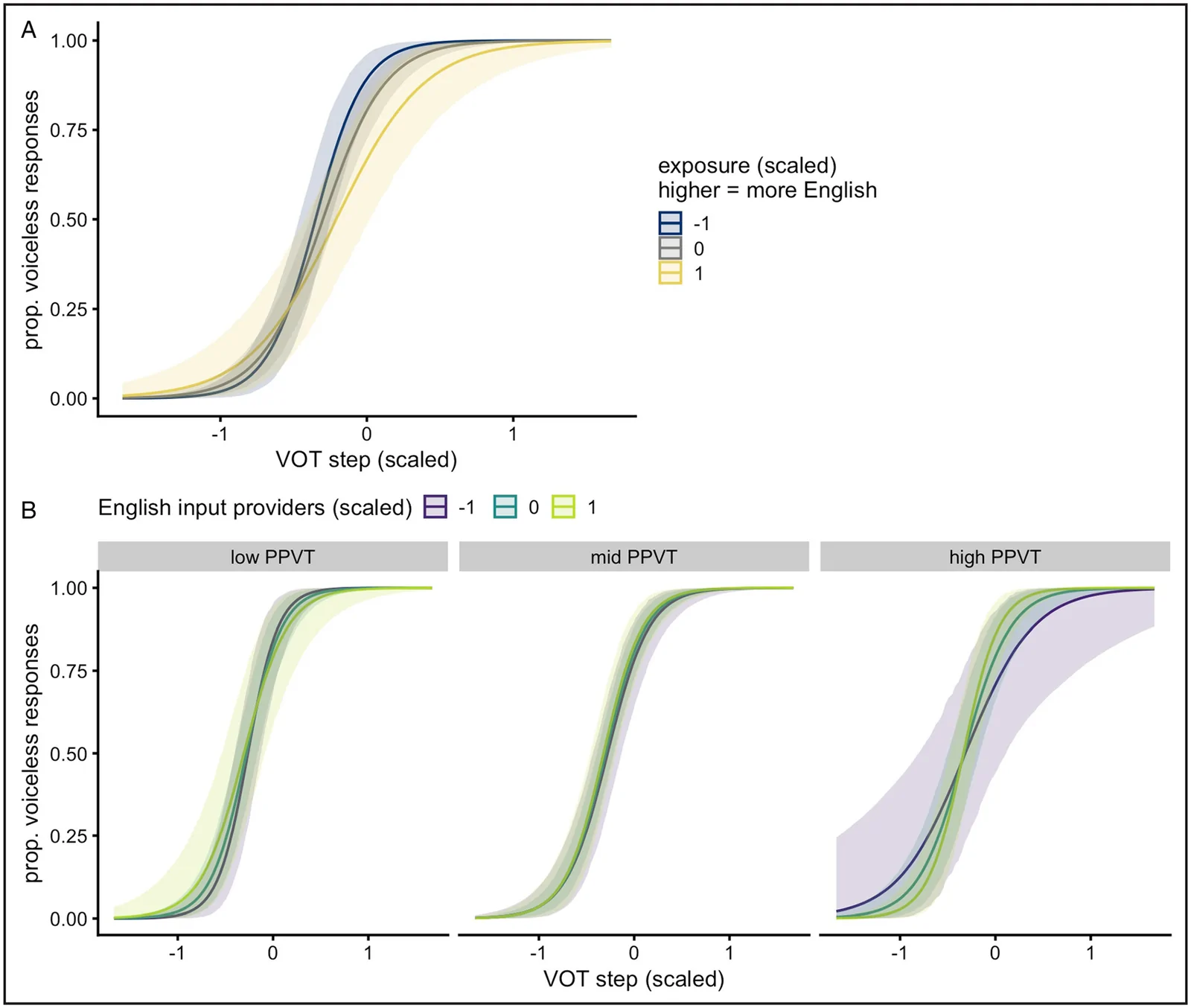
Moderators in bilinguals’ perception of English bilabial and coronal stop stimuli (combined across place of articulation). Panel A shows model predictions for the effect of exposure, marginalized across the other variables. Panel B shows model estimates for the effect of input diversity (line color) and vocabulary (panels), marginalized across the other variables. Ribbons are 95% credible intervals from the model.

Turning to the moderators, there was both a main effect of language exposure (β = −0.71, CrI = [−1.37, −0.02], pd = 98), and a credible interaction between language exposure and continuum step (β = − 1.33, CrI = [−2.54, −0.05], pd = 98). Both of these effects are visualized in Figure 3A. The main effect of exposure can be seen in that increasing exposure scores (i.e., more English exposure) result in more voiced responses in line with more monolingual-like perception patterns, as suggested weakly in the group-level analysis. Thus, more English exposure has influenced stop perception in line with English VOT norms. The interaction between exposure and continuum step provides additional evidence of differences in the categorization slopes of children who hear more English vs. those who hear more Spanish, with the former increasing voiced responses for the more ambiguous items in the middle of the continuum and displaying a higher category boundary (positioned to the right) with respect to the latter.

We also found a credible three-way interaction between continuum step, input diversity (i.e., number of English input providers) and vocabulary (i.e., PPVT score) (β = 1.16, CrI = [0.19,2.24], pd = 100). These effects are visualized in Figure 3B. The figure shows the changing influence of input diversity at different levels of vocabulary. For the smallest vocabulary (low PPVT panel), categorization slopes are steepest with reduced input diversity (purple line). In other words, children with small vocabularies benefit from hearing English input from fewer speakers. The pattern is reversed for larger vocabularies (high PPVT panel): here, increased input diversity (yellow line) results in steeper categorization slopes. The mid-level vocabulary group is intermediate in the sense that no clear difference as a function of input diversity is apparent. Besides these, we did not find credible evidence for other effects or interactions moderating bilinguals’ perception of English stops.

**Figure 4:**
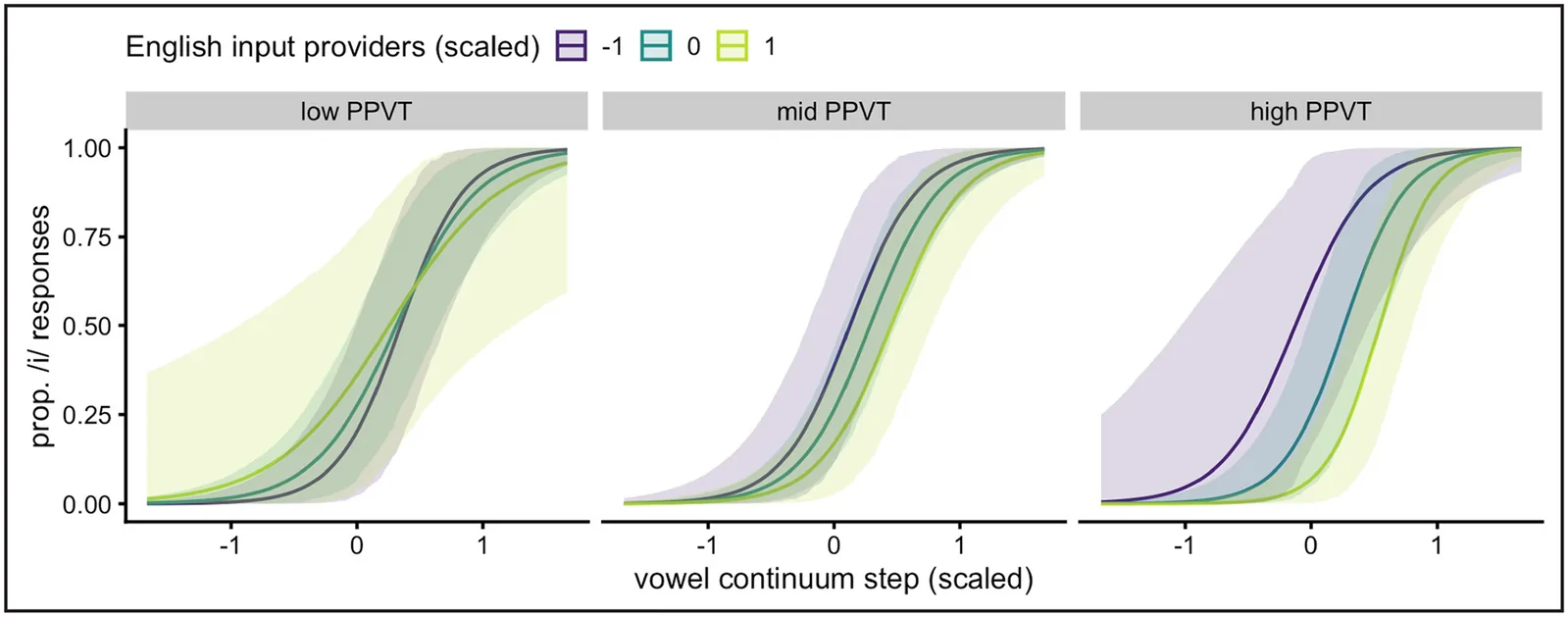
Moderators in bilinguals’ perception of English vowel stimuli, showing model estimates for the effect of input diversity (line color) and vocabulary (panels), marginalized across the other variables. Ribbons are 95% credible intervals from the model.

Turning to the vowel analysis, in addition to an expected effect of continuum step, there was a credible two-way interaction between input diversity and vocabulary (β = −0.97, CrI = [−1.96, −0.00], pd = 98), as shown in Figure 4. This interaction shows that for children with higher vocabulary scores (high PPVT), responses are shifted rightwards when there is more diverse input (green line). For children with mid and low vocabulary scores (mid and low PPVT), the effect diminishes. This can be compared to the stop results, where we can see a similar pattern, but also involving continuum, i.e., *the steepness of the categorization functions*. Here as step is not included, the interaction involves the overall placement of a fit function along the continuum space (i.e., overall log-odds of a particular response), but not the slope of the functions. The directionality of shifting rightwards is the trend observed for monolinguals as compared to bilinguals (Figure 2B); hence the rightward shift may be taken to reflect more monolingual-like response patterns, that is, more /ɪ/ (and fewer /i/ responses), which could relate to the development of the /ɪ/ category, an English-only vowel.

### Moderators of bilingual children’s Spanish stop and vowel perception

**Figure 5:**
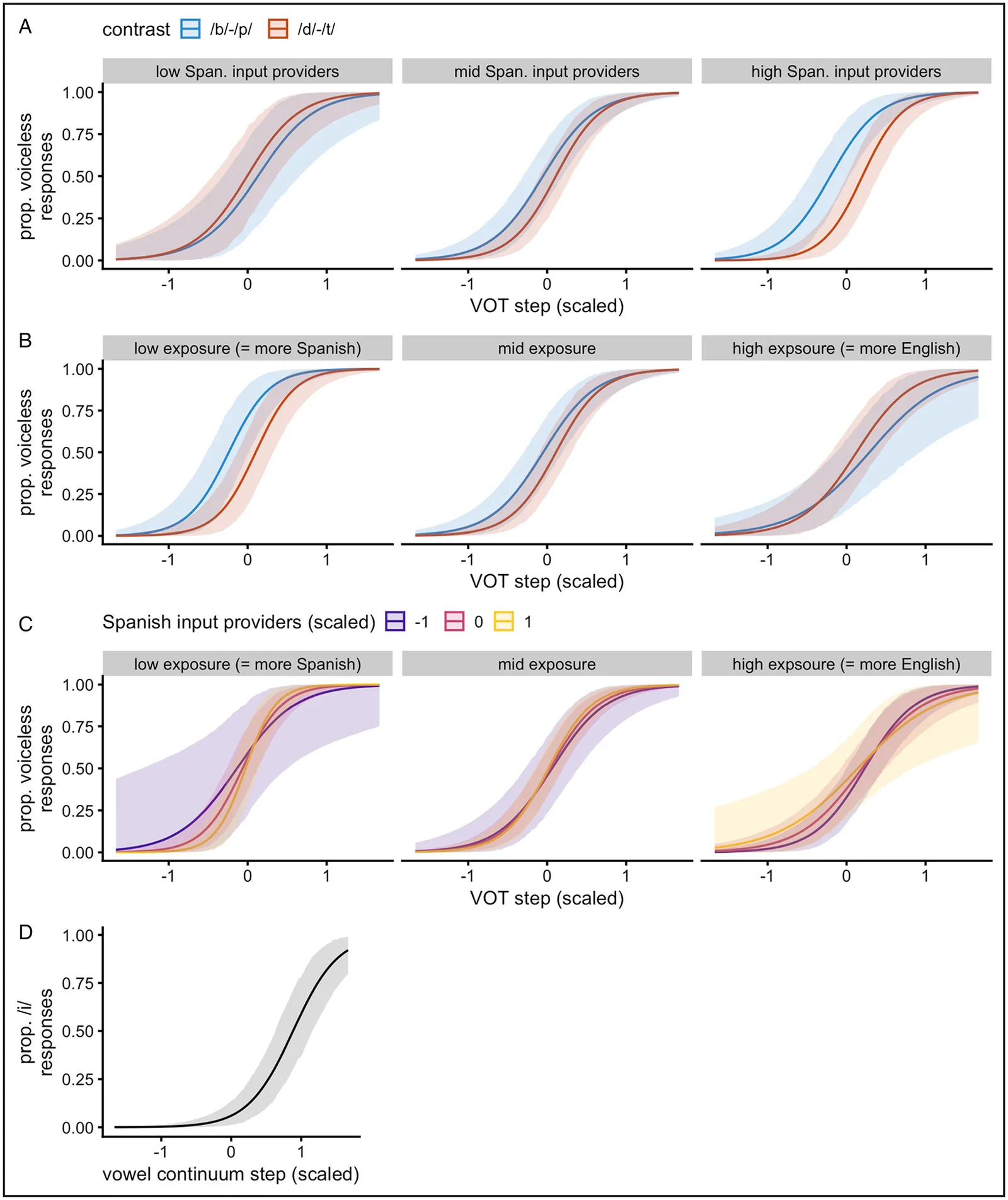
Bilinguals’ categorization of Spanish stops, split by contrast and Spanish input diversity (A), contrast and exposure score (B), and Spanish input diversity and exposure (bilabial and coronal contrasts are combined). (C). Panel D shows the overall vowel responses by continuum step for the Spanish vowel contrast. Ribbons are 95% credible intervals from the model.

In contrast to the models assessing moderators in English, no variable, individually, emerged as having a credible effect in the perception of the Spanish contrasts. In the Spanish stops model, there was a main effect of continuum step (β = 3.28, CrI = [2.42,4.28], pd = 100), showing that, as expected, increasing VOT step increased the log odds of a voiceless response. There were three additional interactions with a pd value in excess of 95. Two of these include contrast interacting with Spanish input diversity (pd = 96) and contrast interacting with language exposure (pd = 96). These are both shown in Figure 5A and 5B. These interactions reflect a separation between place of articulation in terms of overall voiceless responses (analogous to a category boundary) where both diverse Spanish input (Figure 5A, right panel) and more Spanish exposure (Figure 5B, left panel) increase separation between the Spanish bilabial and coronal contrast. However, since each of these CrI narrowly include zero, we should consider these interactions exploratory, as we can only be 96% sure that they exist with this directionality.

The additional credible interaction was between continuum step, Spanish input diversity, and exposure. As shown in Figure 5C, this reflects the fact that for children with more English exposure (right panel), less diverse Spanish input (purple line) increases the steepness of categorization. For children with more Spanish exposure (left panel), the opposite is true, where more diverse Spanish input (yellow line) is more beneficial to the perception of Spanish stops. These results mirror the credible interactions documented in English, although in Spanish, it is exposure, rather than vocabulary, that mediates the role of input diversity on perception.

In the vowel model, in contrast, there was no credible influence of any of the moderator variables, only a credible effect of continuum step (β = 3.13, CrI = [2.12,4.29]) (Figure 5D).

## Discussion

This study aimed to advance our understanding of speech perception in bilingual children by addressing three principal research questions. First, we examined the extent to which Spanish-dominant children’s English stop and vowel perception patterns differed from those of age-, gender-, and SES-matched functional English monolinguals from the same bilingual community. Then, we investigated the extent to which a series of exposure- and proficiency-related variables (language exposure, input diversity, and receptive vocabulary) moderated perception patterns in both the societal and heritage language. To the best of our knowledge, this study is the first to compare the speech perception patterns of Spanish-dominant bilingual children to those of functional monolinguals from the same high-language-contact community. The study is also the first to assess the perception of both vowels and consonants in both the societal and heritage language of Spanish-dominant bilingual children taking into account both child-internal and external variables.

### Bilingual children’s English stop and vowel perception as compared to English monolinguals

The first analysis, which involved a comparison of English stop and vowel perception patterns between bilinguals and monolinguals, showed that bilingual children’s perceptual performance differed only in part from that of monolingual peers from the same community. Indeed, the bilinguals’ perception of the vowel contrast did not differ in any respect from that of the monolinguals, similarly to the participants in Montanari et al. (2024). When it came to stop voicing perception, the bilingual children tended to hear more voiceless stops compared to the monolinguals, in line with Spanish VOT patterns, where positive values (all of the English continuum) are mapped to the voiceless category, whereas English-speaking monolinguals map positive short lag VOT to a voiced category. However, this pattern was marginally significant and a credible—but weak—difference in bilingual vs. monolingual perception was only found for /b-p/, for which monolingual children provided more voiced responses for the more ambiguous items in the middle of the continuum in line with English VOT patterns. These results mirror those of Montanari et al. (2023), who also documented more voiceless responses—and hence cross-linguistic interaction from Spanish—for bilingual children as compared to monolinguals in the perception of the same contrasts. Taken together, these results suggest that experience with Spanish may affect English perceptual performance in bilingual children; however, cross-linguistic interaction may not affect all “similar” sounds as posited by Flege’s SLM-r model (Flege & Bohn, 2021) and Best’s PAM-L2 model (Best & Tyler, 2007), and bilingual–monolingual differences may be more limited in a community where monolinguals may also be exposed to the heritage language and to accented speech.

It is unclear why bilingual–monolingual differences were only found for /b-p/ and not /d-t/. As shown by the two-way interaction, both bilinguals and monolinguals showed more target-like performance with the coronal contrast, which suggests that this contrast is acquired before the bilabial contrast. Montanari et al. (2023) also documented an overall steeper perception of the English coronal contrast for both bilinguals and monolinguals, and age effects only for the bilabial contrast. The authors interpreted this finding as stemming from the larger acoustic difference between /t/ and /d/ than between /p/ and /b/ in English, which makes the coronal contrast more salient (Port & Rotunno, 1979). Indeed, some studies have documented better perception of /d-t/ with respect to /b-p/ in both bilinguals and monolinguals (Williams, 1977; Zlatin & Koenigsknecht, 1975). Moreover, some acquisition theories have proposed the concept of coronal underspecification, according to which the coronal place of articulation is the language universal default place of articulation for phonemes, with the perception and mastery of coronal sounds occurring earlier than that of sounds produced at the bilabial or dorsal place of articulation (Cummings et al., 2020). We speculate, thus, that while both bilingual and monolingual children had perfected their /d-t/ perception, the bilinguals were still in the process of refining their perception of the later-acquired bilabial contrast, showing a different categorization slope with respect to the monolinguals.

### Moderators of bilingual children’s English stop and vowel perception

The analysis of the moderators of bilingual children’s English stop and vowel perception indicated that language exposure, input diversity and receptive vocabulary were related—either individually or collectively—to perceptual performance with both stops and vowels in the societal language. As to stop voicing perception, there was a credible main effect of language exposure, with higher English input resulting in more English-like perceptual performance with English stops. Furthermore, language exposure interacted with continuum step with children hearing more English providing more voiced responses specifically for the more ambiguous items in the middle of the continuum, in line with English VOT patterns. These results provide evidence of differences in the categorization slopes of children with different levels of English exposure, mirroring the results of previous studies that have documented more target-like perceptual abilities with increasing language input (McCarthy et al., 2014; Ramon-Casas et al., 2023; Yu et al., 2019). Interestingly, Montanari et al. (2023) found that language exposure solely moderated perceptual performance in the heritage language, whereas it was not related to perception in English. We speculate that the contradictory results are due to exposure and proficiency differences between the participants of each study: in Montanari et al. (2023), the children were more exposed to English than Spanish and could be assumed to have stronger English skills. In the current study, the children heard more Spanish than English and were dominant in Spanish, as shown by their receptive vocabulary. Since the importance of language exposure wanes at more advanced language skills, when children have reached a critical threshold of proficiency in which only subtler aspects of the language are left to be acquired (Gathercole, 2007), it is possible that our participants had not reached such critical proficiency threshold in the societal language. Taken together, thus, these results suggest that language exposure may be more predictive of perceptual performance in the non-dominant than in the dominant language.

Furthermore, both analyses of the moderators of English stop and vowel perception revealed that receptive vocabulary and input diversity jointly interacted to mediate how well children perceived English stops and vowels. Specifically, diverse input promoted perceptual performance in the context of a large vocabulary, whereas it limited perception in the context of a limited vocabulary size. Put it another way, children benefited from hearing English from multiple speakers (displaying more English-like stop and vowel perceptual patterns) when they had a large English receptive vocabulary. In contrast, children with a small receptive vocabulary showed more target-like perceptual performance only if they heard English from fewer speakers. These results are in line with the findings of Montanari et al. (2024), who found that input diversity benefited vowel perception in the context of high English language exposure, whereas it limited perceptual performance when children received reduced English input. The authors speculated that diverse input may promote perceptual performance at high levels of language exposure and advanced language skills, when children can possibly use input from multiple speakers to *refine* their perceptual categories. Indeed, greater variability in the input may ensure that more of the sound space—and a wider range of stops and vowels—are sampled, ultimately leading to further refined phonological representations and more discrete categorization. In contrast, at low levels of exposure and limited language skills, diverse input—which may also include accented input—may make it harder for children to distinguish between, and form, new perceptual categories, limiting perceptual performance.

Our results advance this proposal by showing that, at least in the societal language (which children may hear from both native and non-native speakers), it is vocabulary knowledge (what children *know*)—even more so than language exposure (what children *hear*)—that mediates the effects of input diversity on perceptual performance: increasing vocabulary size (including knowing phonologically similar words) triggers the need to specify and differentiate phonemic contrasts, leading to better perceptual acuity and more robust and differentiated phonological representations (*lexical restructuring hypothesis*, Metsala & Walley, 1998). In this context, hearing the same word from multiple speakers will allow children to identify inter-speaker variation and differentiate native from non-native sound productions, increasing the word’s quality and contributing to a mental lexicon with highly specific phonological representations (*lexical quality hypothesis*, Perfetti & Hart, 2002). In contrast, smaller vocabularies (with phonologically sparser words) will result in less robust and detailed phonological representations; children with less refined representations will be less likely to recognize between-speaker pronunciation differences and distinguish native and accented productions, showing lower-quality word representations and less sophisticated categorization. In the case of a small vocabulary, then, input that is more homogeneous, that is, less diverse (and assuming non-accented), is more conducive to discrete categorization as children can focus on the speech patterns of a limited number of speakers to refine their phonemic categories. Indeed, studies of children in the early stages of L2 acquisition (when children presumably have small L2 vocabularies) have shown that less variable input may lead to more firmly entrenched storage of speaker-specific phonetic information, improving L2 perception and production outcomes (Giannakopoulou et al., 2017). This is because children find it harder than adults to adapt to multiple talkers, and when hearing a lesser-known language from fewer speakers, they may more readily remember how these speakers produce a certain sound and use this information to shape their own perception and production (Giannakopoulou et al., 2017; Mayr & Montanari, 2015). Hence, receptive vocabulary, taken as a proxy for the extent of phonological detail in representations, may mediate the relation between input diversity and perception, with diverse input being either beneficial or detrimental for perceptual performance based on the learner’s vocabulary size.

### Moderators of bilingual children’s Spanish stop and vowel perception

The analysis of the moderators of bilingual children’s Spanish stop and vowel perception indicated that, unlike in English, none of the exposure- or proficiency-related measures had a main effect, *alone*, on Spanish perceptual performance, suggesting that the children may have been more ahead in their intensification of language-specific perception in Spanish, their dominant language. There were, however, several credible interactions that confirmed the role of language exposure and input diversity on perceptual performance with Spanish stops. As a start, both increased Spanish exposure and input diversity led children to better differentiate bilabial from coronal stop categories in terms of categorical boundary, with the /b-p/ boundary being positioned at lower VOT values as compared to the categorical boundary for /d-t/. This makes sense considering that studies of adult native Spanish speakers show VOT boundary values of 14 ms or less for word-initial bilabial stops (Abramson & Lisker, 1973; Williams, 1977), whereas the category boundary for /d-t/ falls at about 23 ms at word onset (Abramson & Lisker, 1973; Flege & Eefting, 1987). Thus, children who heard more Spanish and had more Spanish interlocutors displayed different categorical boundaries for the /b-p/ and /d-t/ contrasts similarly to Spanish monolingual adults.

The second finding from the analysis of the moderators of Spanish perception was that language exposure (similarly to receptive vocabulary in English) mediated the role of input diversity on perception. Specifically, children with more Spanish exposure showed more Spanish-like perceptual patterns when being exposed to more diverse Spanish input; in contrast, children receiving less Spanish input showed steeper categorization in the context of reduced Spanish input diversity. Although receptive vocabulary may be a better proxy of rich phonological representations, language exposure has been widely shown to be linked to perception (McCarthy et al., 2014; Montanari et al., 2023; Ramon-Casas et al., 2023). Hence, it is possible that while measuring different constructs, both language exposure and receptive vocabulary were indicative of children’s overall proficiency and learning stage. Thus, as shown by Lev-Ari (2018) in child vs. adult computer simulations of speech perception, input variability makes the processing and identification of speech sound categories more challenging at early stages of learning (i.e., in less advanced learners, “child simulations”), while having a facilitatory effect later on when categories already exist and have to be refined (i.e., advanced learners, “adult simulations”). These results are supported by research that finds no facilitative effect of diverse input on speech sound category formation but a facilitative effect of input diversity on phonotactic pattern learning and associative word learning, which develop after categories have been established (Quam & Creel, 2021).

## Conclusions

In summary, this study is the first to compare the English speech perception of Spanish-dominant bilingual children with that of functional monolinguals from the same community, while also documenting their stop and vowel perception abilities in both the societal and heritage language. Our findings highlight bilinguals’ ability to learn—and their flexibility to use—the same acoustic cues (i.e., VOT) differently to perceive sounds in their two languages, showing a dynamic and adaptive system capable of reconfiguring perceptual markers based on the language context. Indeed, the Spanish-dominant bilingual children’s English perceptual performance did not differ substantially from that of English monolingual peers from the same high-language-contact community, suggesting that cross-linguistic interaction—as well as bilingual–monolingual differences—may be more limited in a community where monolinguals also have indirect exposure to the heritage language. These results highlight the need to refine theories of L2 speech perception, which should incorporate the case of child bilinguals who may show asymmetries in their language proficiency, similarly to L2 learners but are young enough to eventually attune to the speech categories of each language. In particular, we need a better account of which specific “acoustically similar sounds” lead to cross-linguistic interaction in bilingual children, for how long interaction effects may be evident, and how increasing exposure to the societal language affects perceptual skills in both the societal and heritage language.

Our study further shows that exposure- and proficiency-related measures moderated perceptual performance in both languages, although there were more credible effects in English. Specifically, greater English exposure led to improved English stop voicing perception, suggesting that for young Spanish-dominant bilinguals who have not yet reached a critical threshold of input, English exposure may significantly influence their ability to perceive speech sounds in the language. Additionally, input diversity interacted with receptive vocabulary in English and language exposure in Spanish to moderate children’s perceptual performance in each language. These findings suggest that both exposure and proficiency-related variables may play a key role in refining language-specific perception, and that input diversity may enhance perceptual performance at advanced stages of learning while hindering it at lower levels. Our results for English—which children may hear from both native and non-native speakers—specifically highlight that increasing vocabulary size coupled with diverse input may particularly help young Spanish-dominant bilinguals identify inter-speaker variation and differentiate native from accented productions, leading to improved lexical quality and more specific and differentiated phonological representations in the societal language.

Our results are exploratory as the study is limited in a number of ways. First, while we designed our study to only compare our bilingual participants’ perception to that of monolinguals in the societal language, the lack of a Spanish monolingual group prevented us from assessing how the bilinguals’ Spanish perception compared to that of Spanish monolinguals. We avoided such comparison due to evident input and language learning environment differences between heritage language speakers and monolinguals in geographically distant sociolinguistic realities (Bayram et al., 2021). Yet, monolingual data in this scenario can be useful to interpret whether bilingual patterns arise from developmental factors or cross-linguistic interaction. Second, our sample was relatively small and did not include an equal number of boys and girls. While our results reveal promising links between speech perception and proficiency- and exposure-related variables, our field is in great need of larger-scale studies to properly model all of the different aspects that moderate perceptual performance in both the heritage (or dominant) and societal (or non-dominant) language in this population. Furthermore, we also only included certain child-internal and external factors as moderators of perceptual performance, and some of our measures were perhaps not sufficiently fine-grained to capture children’s full range of language exposure. Future research should include more sophisticated measures of input quantity (i.e., absolute language exposure) and quality (i.e., level of exposure to native vs. accented speech) and examine the role of other variables on speech perception, including broader sociolinguistic variables such as maternal education and acculturation. Finally, future studies should track bilingual children’s perception from infancy to school-age, documenting changes from age 2, when home language should still be dominant, to age 3, when exposure to English in preschool begins, to the outset of formal schooling in kindergarten at age 5. It is particularly important that future studies be longitudinal because only this methodology can show how increasing exposure to the mainstream culture affects perception in both the societal and heritage language.

## Figures and Tables

**Table 1:** Bilinguals’ and monolinguals’ language exposure, number of English and Spanish input providers, PPVT, and TVIP raw and standard scores.

Measure (*M*, SD)	Bilinguals (*N* = 37)	Monolinguals (*N* = 37)
Language Exposure^a^	2.86 (0.87)	4.7 (0.46)
Input Diversity (English)^b^	3.78 (0.95)	5.08 (0.75)
Input Diversity (Spanish)^b^	4.14 (1.41)	NA
PPVT (raw)^c^	71.05 (29.33)	86.32 (24.6)
PPVT (standard)^d^	91.81 (12.64)	104.62 (15.4)
TVIP (raw)^c^	27.76 (13.86)	NA
TVIP (standard)^d^	103.24 (16.77)	NA

a Measured on a 1 to 5 scale with 1 representing “child hears mostly Spanish,” 2 “child hears more Spanish than English,” 3 “child hears as much Spanish as English,” 4 “child hears more English than Spanish,” and 5 “child hears mostly English.”

b Measured by including multiple input sources among “mother, father, siblings, grandparents, babysitter, teacher, media, and other.”

c Number of correct vocabulary items in the test before reaching ceiling.

d These are based on monolingual norms, with a mean of 100 and standard deviation of 15.

## Data Availability

The data and codes necessary to reproduce the analyses presented here are publicly accessible, as the materials necessary to attempt to replicate the findings can be requested from the first author. Analyses were not pre-registered. Data and code for analysis and visualization are available on the OSF: https://osf.io/w57ar/

## References

[R1] Abramson A, & Lisker L. (1973). Voice-timing perception in Spanish word-initial stops. Journal of Phonetics, 1, 1–8. 10.1016/S0095-4470(19)31372-5

[R2] Allen JS, & Miller JL. (1999). Effects of syllable-initial voicing and speaking rate on the temporal characteristics of monosyllabic words. The Journal of the Acoustical Society of America, 106, 2031–2039. 10.1121/1.427949 10530026

[R3] Baigorri M, Campanelli L, & Levy E. (2019). Perception of American–English vowels by early and late Spanish–English bilinguals. Language and Speech, 62, 681–700. 10.1177/0023830918806933 PMC6561833 30354920

[R4] Bayram F, Kubota M, Luque A, Pascual y Cabo D, & Rothman J. (2021). You can’t fix what is not broken: Contextualizing the imbalance of perceptions about heritage language bilingualism. Frontiers in Education, 6, 628311. 10.3389/feduc.2021.628311.

[R5] Best CT, & Tyler MD. (2007). Nonnative and second-language speech perception: Commonalities and complementarities. In Munro MJ, & Bohn O-S. (Eds.), Second language speech learning: The role of language experience in speech perception and production (pp. 13–34). John Benjamins.

[R6] Bosch L, & Sebastián-Gallés N. (2003). Simultaneous bilingualism and the perception of a language-specific vowel contrast in the first year of life. Language and Speech, 46, 217–243. 10.1177/00238309030460020801 14748445

[R7] Bürkner PC. (2017). Brms: An R package for Bayesian multilevel models using Stan. Journal of Statistical Software, 80, 1–28. 10.18637/jss.v080.i01

[R8] Burnham DK, Earnshaw LJ, & Clark JE. (1991). Development of categorical identification of native and non-native bilabial stops: Infants, children and adults. Journal of Child Language, 18, 231–260. 10.1017/s0305000900011041 1874826

[R9] Bushong W, & Jaeger TF. (2019). Dynamic re-weighting of acoustic and contextual cues in spoken word recognition. The Journal of the Acoustical Society of America, 146, EL135–EL140. 10.1121/10.0019956 PMC7273512 31472578

[R10] Calandruccio L, Beninate I, Oleson J, Miller MK, Leibold LJ, Buss E, & Rodriguez BL. (2021). A simplified approach to quantifying a child’s bilingual language experience. American Journal of Audiology, 30, 769–776. 10.1044/2021_AJA-20-00214 PMC8642093 34310200

[R11] Cummings AE, Ogiela DA, & Wu YC. (2020). Evidence for [coronal] underspecification in typical and atypical phonological development. Frontiers in Human Neuroscience, 14, 580697. 10.3389/fnhum.2020.580697 PMC7782969 33414710

[R12] Dmitrieva O, Llanos F, Shultz AA, & Francis AL. (2015). Phonological status, not voice onset time, determines the acoustic realization of onset f0 as a secondary voicing cue in Spanish and English. Journal of Phonetics, 49, 77–95. 10.1016/j.wocn.2014.12.005

[R13] Dunn DM. (2018). Peabody picture vocabulary test (Fifth Edition (PPVT-5)). Pearson Assessments.

[R14] Dunn LM, Lugo DE, Padilla ER, & Dunn LM. (1986). TVIP: Test de vocabulario en imagenes peabody: Adaptación hispano-americana [Peabody picture vocabulary test: Hispanic-American adaptation]. American Guidance Service.

[R15] Flege J, & Bohn O-S. (2021). The revised Speech Learning Model (SLM-r). In Wayland R. (Ed.), Second language speech learning: Theoretical and empirical progress (pp. 3–83). Cambridge University Press.

[R16] Flege JE, & Eefting W. (1987). Production and perception of English stops by native Spanish speakers. Journal of Phonetics, 15, 67–83. 10.1016/S0095-4470(19)30538-8

[R17] Gathercole VCM. (2007). Miami and North Wales, so far and yet so near: Constructivist account of morpho-syntactic development in bilingual children. International Journal of Bilingual Education and Bilingualism, 10, 224–246. 10.2167/beb442.0

[R18] Giannakopoulou A, Brown H, Clayards M, & Wonnacott E. (2017). High or low? Comparing high and low-variability phonetic training in adult and child second language learners. PeerJ, 5, e3209. 10.7717/peerj.3209 PMC5452958 28584698

[R19] Grosjean F. (2001). The bilingual’s Language modes. In Nicol J. (Ed.), One mind, two languages: Bilingual language processing (pp. 1–22). Blackwell.

[R20] Hazan V, & Barrett S. (2000). The development of phonemic categorization in children aged 6–12. Journal of Phonetics, 28, 377–396. 10.1006/jpho.2000.0121

[R21] Hillenbrand J, Getty LA, Clark MJ, & Wheeler K. (1995). Acoustic characteristics of American English vowels. The Journal of the Acoustical Society of America, 97, 3099–3111. 10.1121/1.411872 7759650

[R22] Horlyck S, Reid A, & Burnham D. (2012). The relationship between learning to read and language-specific speech perception: Maturation versus experience. Scientific Studies of Reading, 16, 218–239. 10.1080/10888438.2010.546460

[R23] Huttenlocher J, Waterfall H, Vasilyeva M, Vevea J, & Hedges LV. (2010). Sources of variability in children’s language growth. Cognitive Psychology, 61, 343–365. 10.1016/j.cogpsych.2010.08.002 PMC2981670 20832781

[R24] Kingston J, Levy J, Rysling A, & Staub A. (2016). Eye movement evidence for an immediate Ganong effect. Journal of Experimental Psychology: Human Perception and Performance, 42, 1969. 10.1037/xhp0000269 27736119

[R25] Kuhl PK, Stevens E, Hayashi A, Deguchi T, Kiritani S, & Iverson P. (2006). Infants show a facilitation effect for native language phonetic perception between 6 and 12 months. Developmental Science, 9, F13–F21. 10.1111/j.1467-7687.2006.00468.x 16472309

[R26] Lev-Ari S. (2018). The influence of social network size on speech perception. The Quarterly Journal of Experimental Psychology, 71, 2249–2260. DOI 10.1177/1747021817739865 30226426

[R27] Macken M, & Barton D. (1980a). The acquisition of the voicing contrast in English: A study of voice onset time in word-initial stop consonants. Journal of Child Language, 7, 41–74. 10.1017/s0305000900007029 7372738

[R28] Macken M, & Barton D. (1980b). The acquisition of the voicing contrast in Spanish: A phonological study of word-initial stop consonants. Journal of Child Language, 7, 433–458. 10.1017/s0305000900002774 6969264

[R29] Makowski D, Ben-Shachar MS, & Lüdecke D. (2019). Bayestestr: Describing effects and their uncertainty, existence and significance within the Bayesian framework. Journal of Open Source Software, 4, 1541. 10.21105/joss.01541

[R30] Makowski D, Ben-Shachar MS, Patil I, & Lüdecke D. (2020). Estimation of model-based predictions, contrasts and means. CRAN.

[R31] Mayr R, & Montanari S. (2015). Cross-linguistic interaction in trilingual phonological development: The role of the input in the acquisition of the voicing contrast. Journal of Child Language, 42, 1006–1035. 10.1017/S0305000914000592 25330782

[R32] Mayr R, Montanari S, Steffman J, & Baghomian M. (2025). Community language exposure affects voice onset time patterns in Spanish-English bilingual children and functional English monolingual children. Bilingualism: Language and Cognition, 28, 1290–1303. 10.1017/S1366728925000045 PMC12373127 40857471

[R33] McCarthy K, Mahon M, Rosen S, & Evans BG. (2014). Speech perception and production by sequential bilingual children: A longitudinal study of voice onset time acquisition. Child Development, 85, 1965–1980. 10.1111/cdev.12275 PMC4282029 25123987

[R34] Metsala JL, & Walley AC. (1998). Spoken vocabulary growth and the segmental restructuring of lexical representations: Precursors to phonemic awareness and early Reading ability. In Metsala JL, & Ehri LC. (Eds.), Word recognition in beginning literacy (pp. 89–120). Lawrence Erlbaum.

[R35] Montanari S, Steffman J, & Mayr R. (2023). Stop voicing perception in the societal and heritage language of Spanish-English bilingual preschoolers: The role of age, input quantity and input diversity. Journal of Phonetics, 101, 101276. 10.1016/j.wocn.2023.101276 PMC11864794 40012735

[R36] Montanari S, Steffman J, & Mayr R. (2024). English vowel perception in Spanish-English bilingual preschoolers: Multiple-talker input is only beneficial for children with high language exposure levels. Journal of Speech, Language, and Hearing Research, 67, 3643–3659. 10.1044/2024_JSLHR-24-00044 PMC11482580 39292920

[R37] Nittrouer S. (2002). Learning to perceive speech: How fricative perception changes, and how it stays the same. The Journal of the Acoustical Society of America, 112, 711–719. 10.1121/1.1496082 PMC3987659 12186050

[R38] Paradis J, & Jia R. (2017). Bilingual children’s long-term outcomes in English as a second language: Language environment factors predict individual differences in catching up to monolinguals. Developmental Science, 20, 1–15. 10.1111/desc.12433 27320539

[R39] Perfetti CA, & Hart L. (2002). The lexical quality hypothesis. In Verhoeven L, Elbro C, & Reitsma P. (Eds.), Precursors of functional literacy (studies in written language and literacy) (pp. 189–213). John Benjamins.

[R40] Place S, & Hoff E. (2011). Properties of dual language exposure that influence 2-year-olds’ proficiency. Child Development, 82, 1834–1849. 10.1111/j.1467-8624.2011.01660.x PMC4144198 22004372

[R41] Place S, & Hoff E. (2016). Effects and noneffects of input in bilingual environments on dual language skills in 2 ½-year-olds. Bilingualism: Language and Cognition, 19, 1023–1041. 10.1017/S1366728915000322

[R42] Polka L, & Bohn OS. (2011). Natural Referent Vowel (NRV) framework: An emerging view of early phonetic development. Journal of Phonetics, 39, 467–478. 10.1016/j.wocn.2010.08.007

[R43] Polka L, & Werker JF. (1994). Developmental changes in perception of nonnative vowel contrasts. Journal of Experimental Psychology: Human Perception and Performance, 20, 421–435. 10.1037/0096-1523.20.2.421 8189202

[R44] Port RF, & Rotunno R. (1979). Relation between voice-onset time and vowel duration. The Journal of the Acoustical Society of America, 66, 654–662. 10.1121/1.383692 489837

[R45] Qualtrics Experiment Management. Provo, UT. Version October 2022. www.qualtrics.com

[R46] Quam C, & Creel S. (2021). Impacts of acoustic-phonetic variability on perceptual development for spoken language: A review. Wiley Interdisciplinary Reviews: Cognitive Science, 12, e1558. 10.1002/wcs.1558 PMC9836025 33660418

[R47] Ramirez NF, Ramirez RR, Clarke M, Taulu S, & Kuhl PK. (2017). Speech discrimination in 11-month-old bilingual and monolingual infants: A magneto-encephalography study. Developmental Science, 20, e12427. 10.1111/desc.12427. 27041494

[R48] Ramon-Casas M, Cortés S, Benet A, Lleó C, & Bosch L. (2023). Connecting perception and production in early Catalan–Spanish bilingual children: Language dominance and quality of input effects. Journal of Child Language, 50, 155–176. 10.1017/S0305000921000787 36503547

[R49] Schertz J, Carbonell K, & Lotto AJ. (2020). Language specificity in phonetic cue weighting: Monolingual and bilingual perception of the stop voicing contrast in English and Spanish. Phonetica, 77, 186–208. 10.1159/000497278 31018217

[R50] Snowling MJ, Lervåg A, Nash HM, & Hulme C. (2019). Longitudinal relationships between speech perception, phonological skills and reading in children at high-risk of dyslexia. Developmental Science, 22, e12723. 10.1111/desc.12723. PMC6492008 30207641

[R51] Sorenson Duncan T, & Paradis J. (2020). Home language environment and children’s second language acquisition: The special status of input from older siblings. Journal of Child Language, 47, 982–1005. 10.1017/S0305000919000977 32223763

[R52] Stager CL, & Werker JF. (1997). Infants listen for more phonetic detail in speech perception than in word-l earning tasks. Nature, 388, 381–382. 10.1038/41102 9237755

[R53] Sundara M. (2024). The speech perception abilities of bilingual infants. In Amengual M. (Ed.), The Cambridge handbook of bilingual phonetics and phonology (pp. 263–284). Cambridge University Press.

[R54] Toscano JC, & McMurray B. (2012). Cue-integration and context effects in speech: Evidence against speaking-rate normalization. Attention, Perception, & Psychophysics, 74, 1284–1301. 10.3758/s13414-012-0306-z PMC3544203 22532385

[R55] Traunmüller H. (1990). Analytical expressions for the tonotopic sensory scale. The Journal of the Acoustical Society of America, 88, 97–100. 10.1121/1.399849

[R56] Vance M, Rosen S, & Coleman M. (2009). Assessing speech perception in young children and relationships with language skills. International Journal of Audiology, 48, 708–717. 10.1080/14992020902930550 19863356

[R57] Walley AC, & Flege JE. (1999). Effect of lexical status on children’s and adults’ perception of native and non-native vowels. Journal of Phonetics, 27, 307–332. 10.1006/jpho.1999.0098

[R58] Werker JF, & Tees RC. (1984). Cross-language speech-perception—evidence for perceptual reorganization during the 1st year of life. Infant Behavior & Development, 7, 49–63. 10.1016/S0163-6383(84)80022-3

[R59] Williams L. (1977). The perception of stop consonant voicing by Spanish-English bilinguals. Perception & Psychophysics, 21, 289–297. 10.3758/BF03199477

[R60] Winn MB. (2019). GUI-based wizard for creating realistic vowel formant continua (v. 38). Retrieved July 27, 2024, from http://www.mattwinn.com/praat/Make_Formant_Continuum_v38.txt.

[R61] Winn MB. (2020). Manipulation of voice onset time in speech stimuli: A tutorial and flexible Praat script. The Journal of the Acoustical Society of America, 147, 852–866. 10.1121/10.0000692 32113256

[R62] Yu Y, Tessel C, Han X, Campanelli L, Vidal N, Gerometta J, Garrido-Nag K, Datta H, & Shafer V. (2019). Neural indices of vowel discrimination in monolingual and bilingual infants and children. Ear and Hearing, 40, 1376–1390. 10.1097/AUD.0000000000000726 PMC6814506 31033699

[R63] Zhang W, & Steffman J. (2025). Attentional influences on cue weighting in vowel perception: Examining prosodic prominence and informational masking. Attention, Perception, & Psychophysics, 87, 2513–2532. 10.3758/s13414-025-03123-5 PMC12568797 40696109

[R64] Zlatin M, & Koenigsknecht R. (1975). Development of the voicing contrast: Perception of stop consonants. Journal of Speech, Language and Hearing Research, 18, 541–553. 10.1044/jshr.1803.541 1186163

